# Diaqua­bis(*N*,*N*′-dibenzyl­ethane-1,2-diamine-κ^2^ *N*,*N*′)nickel(II) dichloride *N*,*N*-dimethyl­formamide solvate

**DOI:** 10.1107/S1600536809046042

**Published:** 2009-11-07

**Authors:** Yu-Fen Liu, Hai-Tao Xia, Da-Qi Wang, Xiao-Lin Gong

**Affiliations:** a Department of Chemical Engineering, Huaihai Institute of Technology, Lianyungang, Jiangsu 222005, People’s Republic of China; b College of Chemistry and Chemical Engineering, Liaocheng University, Shandong 252059, People’s Republic of China

## Abstract

The asymmetric unit of the title complex, [Ni(C_16_H_20_N_2_)_2_(H_2_O)_2_]Cl_2_·C_3_H_7_NO, consists of two Ni^II^ atoms, each lying on an inversion center, two Cl anions, two *N*,*N*′-dibenzyl­ethane-1,2-diamine ligands, two coordinated water mol­ecules and one *N*,*N*-dimethyl­formamide solvent mol­ecule. Each Ni^II^ atom is six-coordinated in a distorted octa­hedral coordination geometry, with the equatorial plane formed by four N atoms and the axial positions occupied by two water mol­ecules. The complex mol­ecules are linked into a chain along [001] by N—H⋯Cl, N—H⋯O and O—H⋯Cl hydrogen bonds. The C atoms and H atoms of the solvent mol­ecule are disordered over two sites in a ratio of 0.52 (2):0.48 (2).

## Related literature

For related structures, see: Xia *et al.* (2007*a* ▶,*b* ▶).
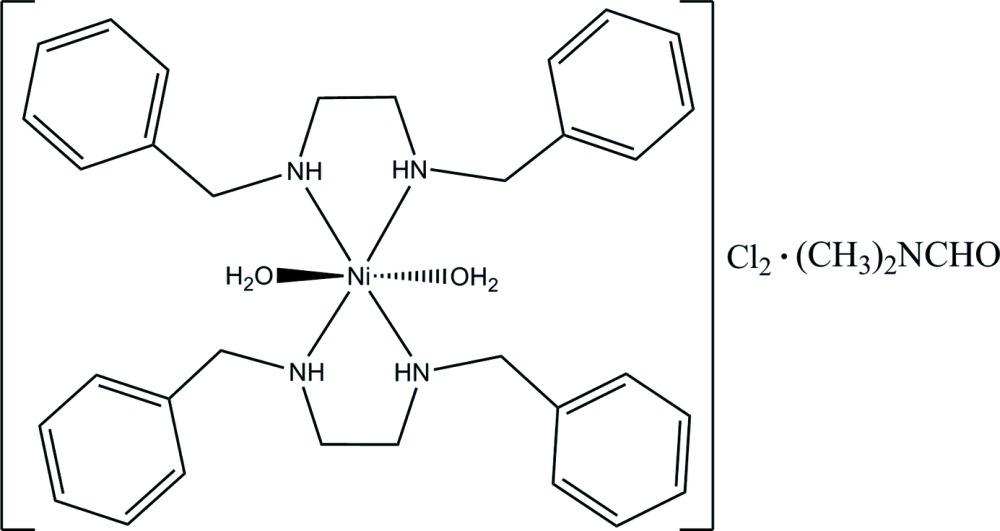


## Experimental

### 

#### Crystal data

[Ni(C_16_H_20_N_2_)_2_(H_2_O)_2_]Cl_2_·C_3_H_7_NO
*M* *_r_* = 719.42
Triclinic, 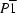

*a* = 11.898 (1) Å
*b* = 12.588 (1) Å
*c* = 14.872 (2) Å
α = 104.769 (2)°
β = 95.368 (1)°
γ = 111.399 (2)°
*V* = 1961.8 (3) Å^3^
*Z* = 2
Mo *K*α radiation
μ = 0.67 mm^−1^
*T* = 298 K
0.42 × 0.40 × 0.35 mm

#### Data collection

Siemens SMART 1000 CCD diffractometer
Absorption correction: multi-scan (*SADABS*; Sheldrick, 1996 ▶) *T*_min_ = 0.767, *T*_max_ = 0.800
10138 measured reflections
6781 independent reflections
4690 reflections with *I* > 2σ(*I*)
*R*_int_ = 0.018

#### Refinement

*R*[*F* ^2^ > 2σ(*F* ^2^)] = 0.066
*wR*(*F* ^2^) = 0.201
*S* = 1.02
6781 reflections
450 parameters
H-atom parameters constrained
Δρ_max_ = 1.15 e Å^−3^
Δρ_min_ = −0.75 e Å^−3^

### 

Data collection: *SMART* (Siemens, 1996 ▶); cell refinement: *SAINT* (Siemens, 1996 ▶); data reduction: *SAINT*; program(s) used to solve structure: *SHELXS97* (Sheldrick, 2008 ▶); program(s) used to refine structure: *SHELXL97* (Sheldrick, 2008 ▶); molecular graphics: *SHELXTL* (Sheldrick, 2008 ▶); software used to prepare material for publication: *SHELXTL*.

## Supplementary Material

Crystal structure: contains datablocks I, global. DOI: 10.1107/S1600536809046042/hy2244sup1.cif

Structure factors: contains datablocks I. DOI: 10.1107/S1600536809046042/hy2244Isup2.hkl

Additional supplementary materials:  crystallographic information; 3D view; checkCIF report

## Figures and Tables

**Table 1:** Selected bond lengths (Å)

Ni1—O1	2.100 (3)
Ni1—N1	2.131 (4)
Ni1—N2	2.143 (4)
Ni2—O2	2.111 (3)
Ni2—N3	2.140 (4)
Ni2—N4	2.130 (4)

**Table 2:** Hydrogen-bond geometry (Å, °)

*D*—H⋯*A*	*D*—H	H⋯*A*	*D*⋯*A*	*D*—H⋯*A*
O1—H1*C*⋯Cl2	0.85	2.26	3.055 (4)	155
O1—H1*D*⋯Cl1^i^	0.85	2.24	3.078 (4)	169
O2—H2*C*⋯Cl1^i^	0.85	2.24	3.071 (4)	168
O2—H2*D*⋯Cl2	0.85	2.19	3.041 (4)	174
N1—H1⋯Cl1^ii^	0.91	2.54	3.444 (4)	171
N2—H2⋯O3^iii^	0.91	2.20	3.102 (8)	169
N3—H3⋯Cl1	0.91	2.55	3.400 (4)	155
N4—H4⋯Cl2^i^	0.91	2.54	3.428 (5)	167

Symmetry codes: (i) 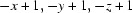; (ii) 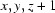; (iii) 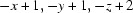.

**Fig. 1.**
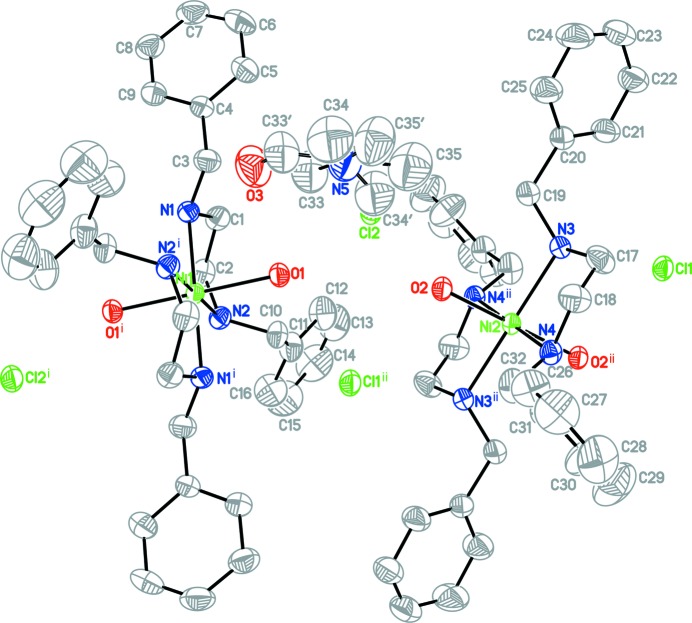
The molecular structure of the title compound. Displacement ellipsoids are drawn at the 30% probability level. [Symmetry codes: (i) 1-x, 1-y, 2-z; (ii) 1-x, 1-y, 1-z.]

## supplementary crystallographic information

### Comment

We have reported the crystal structures of nickel complexes with diamine derivatives (Xia *et al.*, 2007a, b). As a further study of the structures of such complexes, we reported here the crystal structure of the title complex.

The Ni^II^ atom has a slightly distorted octahedral configuration, coordinated by four N atoms from two *N*,*N*'-dibenzylethane-1,2-diamine ligands and two O atoms of two water molecules (Fig. 1 and Table 1). The four N atoms occupy the equatorial positions and O atoms occupy the axial positions. The complex molecules are linked into a chain along the [0 0 1] direction by N—H···Cl and O—H···Cl hydrogen bonds (Table 2). The disordered N,N-dimethylformamide (DMF) solvent molecule is attached to the complex molecule through an N—H···O hydrogen bond.

### Experimental

A solution of N,N'-dibenzylethane-1,2-diamine (1 mmol) in ethanol (20 ml) and a solution of nickel(II) chloride (1 mmol) in ethanol (10 ml) was mixed and the reaction mixture was stirred for 4 h at 328 K. The solution was then cooled slowly to room temperature and filtered. Blue crystals suitable for X-ray diffraction were obtained by evaporation of the ethanol solution.

### Refinement

H atoms were located in difference Fourier maps and refined as riding atoms, with C—H = 0.93 (aryl, formyl), 0.97 (methylene) and 0.96 Å (methyl), N—H = 0.91 Å and O—H = 0.85 Å and with *U*_iso_(H) = 1.2(1.5 for methyl)*U*_eq_(C,N,O). The C and H atoms in the DMF molecule were found to be disordered over two site. The occupancy factors of the two sites were refined to 0.52 (2) and 0.48 (2). The highest residual electron density was found 0.06 Å from C27 and the deepest hole 0.17 Å from C29.

### Crystal data

**Table:**

[Ni(C_16_H_20_N_2_)_2_(H_2_O)_2_]Cl_2_·C_3_H_7_NO	*Z* = 2
*M**_r_* = 719.42	*F*(000) = 764
Triclinic, *P*1	*D*_x_ = 1.218 Mg m^−^^3^
Hall symbol: -P 1	Mo *K*α radiation, λ = 0.71073 Å
*a* = 11.898 (1) Å	Cell parameters from 3426 reflections
*b* = 12.588 (1) Å	θ = 2.4–28.9°
*c* = 14.872 (2) Å	µ = 0.67 mm^−^^1^
α = 104.769 (2)°	*T* = 298 K
β = 95.368 (1)°	Block, blue
γ = 111.399 (2)°	0.42 × 0.40 × 0.35 mm
*V* = 1961.8 (3) Å^3^	

### Data collection

**Table:**

Siemens SMART 1000 CCD diffractometer	6781 independent reflections
Radiation source: fine-focus sealed tube	4690 reflections with *I* > 2σ(*I*)
graphite	*R*_int_ = 0.018
φ and ω scans	θ_max_ = 25.0°, θ_min_ = 1.5°
Absorption correction: multi-scan (*SADABS*; Sheldrick, 1996)	*h* = −14→12
*T*_min_ = 0.767, *T*_max_ = 0.800	*k* = −14→14
10138 measured reflections	*l* = −15→17

### Refinement

**Table:**

Refinement on *F*^2^	Primary atom site location: structure-invariant direct methods
Least-squares matrix: full	Secondary atom site location: difference Fourier map
*R*[*F*^2^ > 2σ(*F*^2^)] = 0.066	Hydrogen site location: inferred from neighbouring sites
*wR*(*F*^2^) = 0.201	H-atom parameters constrained
*S* = 1.02	*w* = 1/[σ^2^(*F*_o_^2^) + (0.0878*P*)^2^ + 5.0932*P*] where *P* = (*F*_o_^2^ + 2*F*_c_^2^)/3
6781 reflections	(Δ/σ)_max_ = 0.001
450 parameters	Δρ_max_ = 1.15 e Å^−^^3^
0 restraints	Δρ_min_ = −0.74 e Å^−^^3^

### Fractional atomic coordinates and isotropic or equivalent isotropic displacement parameters (Å^2^)

**Table:**

	*x*	*y*	*z*	*U*_iso_*/*U*_eq_	Occ. (<1)
Ni1	0.5000	0.5000	1.0000	0.0360 (2)	
Ni2	0.5000	0.5000	0.5000	0.0373 (2)	
Cl1	0.61243 (14)	0.70108 (13)	0.30429 (9)	0.0584 (4)	
Cl2	0.68699 (16)	0.69555 (13)	0.79097 (10)	0.0709 (5)	
N1	0.6479 (4)	0.6692 (4)	1.0730 (3)	0.0487 (10)	
H1	0.6481	0.6822	1.1361	0.058*	
N2	0.4030 (4)	0.6078 (4)	0.9772 (3)	0.0523 (11)	
H2	0.3301	0.5789	0.9959	0.063*	
N3	0.6784 (4)	0.5756 (4)	0.4682 (3)	0.0459 (10)	
H3	0.6763	0.6311	0.4398	0.055*	
N4	0.4979 (4)	0.3356 (4)	0.4135 (3)	0.0465 (10)	
H4	0.4540	0.3180	0.3543	0.056*	
N5	0.8816 (7)	0.4000 (8)	0.7989 (6)	0.113 (2)	
O1	0.5688 (3)	0.4934 (3)	0.8746 (2)	0.0506 (9)	
H1C	0.5980	0.5626	0.8676	0.061*	
H1D	0.5127	0.4452	0.8263	0.061*	
O2	0.5814 (3)	0.4709 (3)	0.6187 (2)	0.0479 (8)	
H2C	0.5255	0.4151	0.6315	0.057*	
H2D	0.6057	0.5340	0.6663	0.057*	
O3	0.8337 (6)	0.4541 (7)	0.9394 (5)	0.118 (2)	
C1	0.6111 (6)	0.7603 (5)	1.0454 (4)	0.0594 (15)	
H1A	0.6266	0.7620	0.9828	0.071*	
H1B	0.6594	0.8395	1.0904	0.071*	
C2	0.4762 (6)	0.7274 (5)	1.0450 (4)	0.0616 (15)	
H2A	0.4614	0.7281	1.1081	0.074*	
H2B	0.4514	0.7860	1.0274	0.074*	
C3	0.7735 (6)	0.6875 (6)	1.0621 (5)	0.0678 (17)	
H3A	0.7754	0.6708	0.9950	0.081*	
H3B	0.7951	0.6297	1.0839	0.081*	
C4	0.8705 (5)	0.8117 (5)	1.1149 (4)	0.0595 (15)	
C5	0.9575 (6)	0.8730 (6)	1.0714 (5)	0.079 (2)	
H5	0.9558	0.8375	1.0079	0.095*	
C6	1.0475 (7)	0.9859 (7)	1.1193 (6)	0.091 (2)	
H6	1.1064	1.0247	1.0884	0.109*	
C7	1.0504 (7)	1.0406 (7)	1.2117 (6)	0.084 (2)	
H7	1.1098	1.1176	1.2433	0.101*	
C8	0.9665 (6)	0.9831 (6)	1.2580 (5)	0.0779 (19)	
H8	0.9681	1.0198	1.3212	0.093*	
C9	0.8785 (6)	0.8689 (6)	1.2093 (5)	0.0721 (18)	
H9	0.8225	0.8289	1.2415	0.087*	
C10	0.3726 (6)	0.6090 (6)	0.8795 (4)	0.0608 (15)	
H10A	0.3191	0.5279	0.8402	0.073*	
H10B	0.4482	0.6323	0.8556	0.073*	
C11	0.3108 (7)	0.6912 (7)	0.8681 (5)	0.0738 (18)	
C12	0.3740 (8)	0.7947 (7)	0.8463 (5)	0.087 (2)	
H12	0.4542	0.8104	0.8376	0.105*	
C13	0.3246 (10)	0.8759 (8)	0.8368 (6)	0.105 (3)	
H13	0.3690	0.9444	0.8215	0.127*	
C14	0.2083 (11)	0.8505 (9)	0.8508 (7)	0.111 (3)	
H14	0.1736	0.9048	0.8466	0.134*	
C15	0.1388 (10)	0.7483 (10)	0.8709 (7)	0.114 (3)	
H15	0.0579	0.7326	0.8776	0.137*	
C16	0.1923 (8)	0.6681 (8)	0.8810 (6)	0.093 (2)	
H16	0.1477	0.5998	0.8964	0.111*	
C17	0.6887 (6)	0.4775 (6)	0.3940 (5)	0.0690 (17)	
H17A	0.6532	0.4758	0.3320	0.083*	
H17B	0.7752	0.4935	0.3959	0.083*	
C18	0.6255 (6)	0.3591 (6)	0.4070 (5)	0.0700 (18)	
H18A	0.6277	0.2969	0.3538	0.084*	
H18B	0.6687	0.3565	0.4645	0.084*	
C19	0.7840 (6)	0.6380 (6)	0.5480 (4)	0.0681 (17)	
H19A	0.7954	0.5786	0.5742	0.082*	
H19B	0.7632	0.6910	0.5969	0.082*	
C20	0.9051 (5)	0.7107 (6)	0.5289 (4)	0.0619 (15)	
C21	0.9161 (6)	0.7821 (7)	0.4713 (5)	0.0765 (19)	
H21	0.8450	0.7824	0.4397	0.092*	
C22	1.0293 (7)	0.8535 (7)	0.4590 (6)	0.088 (2)	
H22	1.0333	0.9006	0.4193	0.105*	
C23	1.1351 (7)	0.8556 (8)	0.5043 (6)	0.094 (2)	
H23	1.2113	0.9020	0.4945	0.113*	
C24	1.1279 (7)	0.7882 (8)	0.5646 (6)	0.095 (2)	
H24	1.1994	0.7909	0.5981	0.115*	
C25	1.0129 (6)	0.7157 (7)	0.5754 (5)	0.082 (2)	
H25	1.0090	0.6692	0.6156	0.098*	
C26	0.4477 (7)	0.2302 (5)	0.4460 (5)	0.0692 (17)	
H26A	0.3652	0.2195	0.4555	0.083*	
H26B	0.4979	0.2469	0.5073	0.083*	
C27	0.4410 (9)	0.1149 (6)	0.3818 (6)	0.088 (2)	
C28	0.3607 (10)	0.0594 (7)	0.2950 (7)	0.109 (3)	
H28	0.3129	0.0956	0.2734	0.131*	
C29	0.3520 (11)	−0.0524 (8)	0.2396 (7)	0.119 (3)	
H29	0.2956	−0.0935	0.1820	0.142*	
C30	0.4289 (10)	−0.1003 (8)	0.2721 (7)	0.108 (3)	
H30	0.4233	−0.1743	0.2347	0.130*	
C31	0.5115 (10)	−0.0458 (8)	0.3553 (7)	0.108 (3)	
H31	0.5633	−0.0793	0.3752	0.130*	
C32	0.5149 (9)	0.0617 (7)	0.4086 (6)	0.102 (3)	
H32	0.5707	0.1011	0.4666	0.122*	
C33	0.8308 (18)	0.4586 (19)	0.8564 (15)	0.111 (8)	0.52 (2)
H33	0.7930	0.5032	0.8351	0.133*	0.52 (2)
C34	0.945 (3)	0.326 (3)	0.814 (2)	0.125 (8)	0.52 (2)
H34A	0.9647	0.3377	0.8813	0.187*	0.52 (2)
H34B	1.0198	0.3480	0.7907	0.187*	0.52 (2)
H34C	0.8930	0.2431	0.7819	0.187*	0.52 (2)
C35	0.877 (2)	0.425 (2)	0.7071 (17)	0.137 (9)	0.52 (2)
H35A	0.8074	0.3613	0.6612	0.206*	0.52 (2)
H35B	0.9513	0.4304	0.6853	0.206*	0.52 (2)
H35C	0.8680	0.4996	0.7153	0.206*	0.52 (2)
C33'	0.8850 (18)	0.400 (2)	0.8893 (16)	0.109 (8)	0.48 (2)
H33'	0.9258	0.3605	0.9140	0.130*	0.48 (2)
C34'	0.803 (2)	0.441 (2)	0.7512 (16)	0.119 (8)	0.48 (2)
H34D	0.7266	0.4207	0.7722	0.178*	0.48 (2)
H34E	0.7880	0.4038	0.6838	0.178*	0.48 (2)
H34F	0.8431	0.5268	0.7656	0.178*	0.48 (2)
C35'	0.946 (3)	0.329 (3)	0.751 (2)	0.128 (9)	0.48 (2)
H35D	1.0249	0.3525	0.7903	0.193*	0.48 (2)
H35E	0.9559	0.3417	0.6912	0.193*	0.48 (2)
H35F	0.8974	0.2450	0.7411	0.193*	0.48 (2)

### Atomic displacement parameters (Å^2^)

**Table:**

	*U*^11^	*U*^22^	*U*^33^	*U*^12^	*U*^13^	*U*^23^
Ni1	0.0397 (5)	0.0367 (5)	0.0311 (4)	0.0144 (4)	0.0077 (3)	0.0110 (4)
Ni2	0.0429 (5)	0.0381 (5)	0.0319 (4)	0.0171 (4)	0.0097 (4)	0.0107 (4)
Cl1	0.0708 (9)	0.0528 (8)	0.0422 (7)	0.0125 (7)	0.0107 (6)	0.0183 (6)
Cl2	0.0868 (11)	0.0541 (9)	0.0448 (8)	−0.0003 (8)	0.0074 (7)	0.0159 (6)
N1	0.050 (3)	0.047 (3)	0.047 (2)	0.016 (2)	0.013 (2)	0.013 (2)
N2	0.059 (3)	0.055 (3)	0.051 (3)	0.030 (2)	0.012 (2)	0.021 (2)
N3	0.046 (2)	0.051 (3)	0.048 (2)	0.023 (2)	0.015 (2)	0.020 (2)
N4	0.054 (3)	0.044 (2)	0.044 (2)	0.023 (2)	0.015 (2)	0.0112 (19)
N5	0.109 (6)	0.158 (8)	0.100 (6)	0.083 (6)	0.037 (5)	0.038 (5)
O1	0.058 (2)	0.052 (2)	0.0403 (19)	0.0182 (18)	0.0159 (17)	0.0158 (16)
O2	0.054 (2)	0.051 (2)	0.0382 (18)	0.0191 (17)	0.0113 (16)	0.0163 (16)
O3	0.109 (5)	0.151 (6)	0.109 (5)	0.064 (5)	0.038 (4)	0.039 (4)
C1	0.065 (4)	0.050 (3)	0.060 (3)	0.020 (3)	0.009 (3)	0.018 (3)
C2	0.073 (4)	0.062 (4)	0.060 (4)	0.040 (3)	0.018 (3)	0.018 (3)
C3	0.060 (4)	0.068 (4)	0.068 (4)	0.019 (3)	0.018 (3)	0.017 (3)
C4	0.053 (3)	0.056 (4)	0.062 (4)	0.012 (3)	0.011 (3)	0.023 (3)
C5	0.068 (4)	0.075 (5)	0.076 (4)	0.008 (4)	0.017 (4)	0.020 (4)
C6	0.070 (5)	0.078 (5)	0.095 (6)	−0.004 (4)	0.018 (4)	0.027 (4)
C7	0.067 (5)	0.066 (5)	0.092 (5)	0.003 (4)	−0.002 (4)	0.022 (4)
C8	0.072 (4)	0.063 (4)	0.076 (5)	0.010 (4)	0.000 (4)	0.016 (4)
C9	0.066 (4)	0.063 (4)	0.069 (4)	0.006 (3)	0.009 (3)	0.021 (3)
C10	0.074 (4)	0.069 (4)	0.056 (3)	0.041 (3)	0.018 (3)	0.028 (3)
C11	0.085 (5)	0.084 (5)	0.074 (4)	0.052 (4)	0.020 (4)	0.034 (4)
C12	0.104 (6)	0.082 (5)	0.091 (5)	0.048 (5)	0.017 (4)	0.037 (4)
C13	0.122 (8)	0.096 (6)	0.108 (7)	0.049 (6)	0.013 (6)	0.044 (5)
C14	0.120 (8)	0.104 (7)	0.121 (7)	0.063 (7)	0.006 (6)	0.035 (6)
C15	0.107 (7)	0.118 (8)	0.124 (8)	0.061 (7)	0.016 (6)	0.027 (6)
C16	0.094 (6)	0.096 (6)	0.102 (6)	0.053 (5)	0.013 (5)	0.035 (5)
C17	0.063 (4)	0.068 (4)	0.072 (4)	0.025 (3)	0.027 (3)	0.013 (3)
C18	0.063 (4)	0.063 (4)	0.077 (4)	0.029 (3)	0.022 (3)	0.003 (3)
C19	0.055 (4)	0.082 (5)	0.064 (4)	0.019 (3)	0.013 (3)	0.032 (3)
C20	0.050 (3)	0.075 (4)	0.065 (4)	0.026 (3)	0.014 (3)	0.028 (3)
C21	0.055 (4)	0.090 (5)	0.081 (5)	0.019 (4)	0.009 (3)	0.038 (4)
C22	0.065 (5)	0.098 (6)	0.089 (5)	0.014 (4)	0.012 (4)	0.043 (4)
C23	0.060 (5)	0.109 (6)	0.099 (6)	0.013 (4)	0.018 (4)	0.037 (5)
C24	0.059 (5)	0.111 (7)	0.106 (6)	0.024 (4)	0.005 (4)	0.037 (5)
C25	0.061 (4)	0.093 (5)	0.089 (5)	0.023 (4)	0.008 (4)	0.040 (4)
C26	0.093 (5)	0.048 (4)	0.066 (4)	0.029 (3)	0.024 (4)	0.014 (3)
C27	0.130 (7)	0.059 (4)	0.092 (5)	0.058 (5)	0.027 (5)	0.018 (4)
C28	0.142 (8)	0.073 (5)	0.107 (7)	0.051 (6)	0.017 (6)	0.012 (5)
C29	0.150 (9)	0.080 (6)	0.109 (7)	0.043 (6)	0.018 (6)	0.010 (5)
C30	0.146 (9)	0.069 (5)	0.108 (7)	0.053 (6)	0.030 (6)	0.010 (5)
C31	0.142 (8)	0.072 (5)	0.111 (7)	0.054 (6)	0.025 (6)	0.015 (5)
C32	0.137 (8)	0.067 (5)	0.105 (6)	0.053 (5)	0.023 (6)	0.018 (4)
C33	0.108 (14)	0.147 (17)	0.105 (16)	0.078 (13)	0.030 (12)	0.041 (13)
C34	0.122 (17)	0.16 (2)	0.12 (2)	0.083 (16)	0.023 (17)	0.045 (19)
C35	0.129 (19)	0.17 (2)	0.123 (18)	0.079 (17)	0.026 (14)	0.037 (16)
C33'	0.107 (15)	0.148 (19)	0.106 (16)	0.085 (14)	0.034 (12)	0.041 (13)
C34'	0.112 (17)	0.148 (19)	0.108 (17)	0.066 (15)	0.025 (13)	0.039 (14)
C35'	0.128 (19)	0.16 (2)	0.11 (2)	0.081 (18)	0.024 (18)	0.037 (19)

### Geometric parameters (Å, °)

**Table:**

Ni1—O1	2.100 (3)	C12—H12	0.9300
Ni1—N1	2.131 (4)	C13—C14	1.350 (13)
Ni1—N2	2.143 (4)	C13—H13	0.9300
Ni2—O2	2.111 (3)	C14—C15	1.377 (13)
Ni2—N3	2.140 (4)	C14—H14	0.9300
Ni2—N4	2.130 (4)	C15—C16	1.405 (12)
N1—C3	1.458 (7)	C15—H15	0.9300
N1—C1	1.499 (7)	C16—H16	0.9300
N1—H1	0.9100	C17—C18	1.476 (9)
N2—C2	1.465 (7)	C17—H17A	0.9700
N2—C10	1.469 (7)	C17—H17B	0.9700
N2—H2	0.9100	C18—H18A	0.9700
N3—C19	1.460 (7)	C18—H18B	0.9700
N3—C17	1.482 (7)	C19—C20	1.497 (8)
N3—H3	0.9100	C19—H19A	0.9700
N4—C18	1.455 (7)	C19—H19B	0.9700
N4—C26	1.467 (7)	C20—C25	1.371 (9)
N4—H4	0.9100	C20—C21	1.373 (9)
N5—C33	1.32 (2)	C21—C22	1.379 (9)
N5—C33'	1.34 (2)	C21—H21	0.9300
N5—C34'	1.43 (2)	C22—C23	1.359 (11)
N5—C34	1.44 (2)	C22—H22	0.9300
N5—C35'	1.47 (3)	C23—C24	1.372 (11)
N5—C35	1.48 (2)	C23—H23	0.9300
O1—H1C	0.8500	C24—C25	1.391 (10)
O1—H1D	0.8500	C24—H24	0.9300
O2—H2C	0.8500	C25—H25	0.9300
O2—H2D	0.8500	C26—C27	1.490 (9)
O3—C33'	1.229 (19)	C26—H26A	0.9700
O3—C33	1.25 (2)	C26—H26B	0.9700
C1—C2	1.504 (8)	C27—C32	1.372 (11)
C1—H1A	0.9700	C27—C28	1.374 (11)
C1—H1B	0.9700	C28—C29	1.398 (11)
C2—H2A	0.9700	C28—H28	0.9300
C2—H2B	0.9700	C29—C30	1.380 (13)
C3—C4	1.510 (8)	C29—H29	0.9300
C3—H3A	0.9700	C30—C31	1.347 (12)
C3—H3B	0.9700	C30—H30	0.9300
C4—C5	1.372 (9)	C31—C32	1.367 (10)
C4—C9	1.382 (9)	C31—H31	0.9300
C5—C6	1.380 (10)	C32—H32	0.9300
C5—H5	0.9300	C33—H33	0.9300
C6—C7	1.363 (10)	C34—H34A	0.9600
C6—H6	0.9300	C34—H34B	0.9600
C7—C8	1.360 (10)	C34—H34C	0.9600
C7—H7	0.9300	C35—H35A	0.9600
C8—C9	1.384 (9)	C35—H35B	0.9600
C8—H8	0.9300	C35—H35C	0.9600
C9—H9	0.9300	C33'—H33'	0.9300
C10—C11	1.505 (8)	C34'—H34D	0.9600
C10—H10A	0.9700	C34'—H34E	0.9600
C10—H10B	0.9700	C34'—H34F	0.9600
C11—C16	1.375 (11)	C35'—H35D	0.9600
C11—C12	1.383 (10)	C35'—H35E	0.9600
C12—C13	1.381 (11)	C35'—H35F	0.9600
			
O1—Ni1—O1^i^	180.000 (2)	C8—C9—H9	118.6
O1—Ni1—N1^i^	88.91 (15)	N2—C10—C11	115.3 (5)
O1^i^—Ni1—N1^i^	91.09 (15)	N2—C10—H10A	108.4
O1—Ni1—N1	91.09 (15)	C11—C10—H10A	108.4
O1^i^—Ni1—N1	88.91 (15)	N2—C10—H10B	108.4
N1^i^—Ni1—N1	180.0 (2)	C11—C10—H10B	108.4
O1—Ni1—N2^i^	84.86 (15)	H10A—C10—H10B	107.5
O1^i^—Ni1—N2^i^	95.14 (15)	C16—C11—C12	118.1 (7)
N1^i^—Ni1—N2^i^	83.75 (17)	C16—C11—C10	121.7 (7)
N1—Ni1—N2^i^	96.25 (17)	C12—C11—C10	120.2 (7)
O1—Ni1—N2	95.14 (15)	C13—C12—C11	123.6 (8)
O1^i^—Ni1—N2	84.86 (15)	C13—C12—H12	118.2
N1^i^—Ni1—N2	96.25 (17)	C11—C12—H12	118.2
N1—Ni1—N2	83.75 (17)	C14—C13—C12	116.5 (9)
N2^i^—Ni1—N2	180.0 (2)	C14—C13—H13	121.8
O2^ii^—Ni2—O2	180.000 (1)	C12—C13—H13	121.8
O2^ii^—Ni2—N4	91.57 (15)	C13—C14—C15	123.2 (9)
O2—Ni2—N4	88.43 (15)	C13—C14—H14	118.4
O2^ii^—Ni2—N4^ii^	88.43 (15)	C15—C14—H14	118.4
O2—Ni2—N4^ii^	91.57 (15)	C14—C15—C16	118.8 (10)
N4—Ni2—N4^ii^	180.000 (1)	C14—C15—H15	120.6
O2^ii^—Ni2—N3	90.50 (15)	C16—C15—H15	120.6
O2—Ni2—N3	89.50 (15)	C11—C16—C15	119.7 (9)
N4—Ni2—N3	83.51 (16)	C11—C16—H16	120.1
N4^ii^—Ni2—N3	96.49 (16)	C15—C16—H16	120.1
O2^ii^—Ni2—N3^ii^	89.50 (15)	C18—C17—N3	112.2 (5)
O2—Ni2—N3^ii^	90.50 (15)	C18—C17—H17A	109.2
N4—Ni2—N3^ii^	96.49 (16)	N3—C17—H17A	109.2
N4^ii^—Ni2—N3^ii^	83.51 (16)	C18—C17—H17B	109.2
N3—Ni2—N3^ii^	180.000 (1)	N3—C17—H17B	109.2
C3—N1—C1	111.2 (5)	H17A—C17—H17B	107.9
C3—N1—Ni1	119.6 (4)	N4—C18—C17	111.3 (5)
C1—N1—Ni1	105.4 (3)	N4—C18—H18A	109.4
C3—N1—H1	106.6	C17—C18—H18A	109.4
C1—N1—H1	106.6	N4—C18—H18B	109.4
Ni1—N1—H1	106.6	C17—C18—H18B	109.4
C2—N2—C10	113.5 (5)	H18A—C18—H18B	108.0
C2—N2—Ni1	105.1 (3)	N3—C19—C20	117.5 (5)
C10—N2—Ni1	117.4 (3)	N3—C19—H19A	107.9
C2—N2—H2	106.8	C20—C19—H19A	107.9
C10—N2—H2	106.8	N3—C19—H19B	107.9
Ni1—N2—H2	106.8	C20—C19—H19B	107.9
C19—N3—C17	114.1 (5)	H19A—C19—H19B	107.2
C19—N3—Ni2	117.3 (3)	C25—C20—C21	116.5 (6)
C17—N3—Ni2	105.8 (3)	C25—C20—C19	120.1 (6)
C19—N3—H3	106.3	C21—C20—C19	123.1 (6)
C17—N3—H3	106.3	C20—C21—C22	122.0 (7)
Ni2—N3—H3	106.3	C20—C21—H21	119.0
C18—N4—C26	108.8 (5)	C22—C21—H21	119.0
C18—N4—Ni2	106.0 (3)	C23—C22—C21	120.6 (7)
C26—N4—Ni2	117.2 (3)	C23—C22—H22	119.7
C18—N4—H4	108.2	C21—C22—H22	119.7
C26—N4—H4	108.2	C22—C23—C24	119.0 (7)
Ni2—N4—H4	108.2	C22—C23—H23	120.5
C33—N5—C33'	57.9 (12)	C24—C23—H23	120.5
C33—N5—C34'	66.3 (13)	C23—C24—C25	119.7 (7)
C33'—N5—C34'	123.1 (14)	C23—C24—H24	120.2
C33—N5—C34	130.7 (16)	C25—C24—H24	120.2
C33'—N5—C34	72.8 (14)	C20—C25—C24	122.1 (7)
C34'—N5—C34	159.6 (16)	C20—C25—H25	118.9
C33—N5—C35'	168.7 (17)	C24—C25—H25	118.9
C33'—N5—C35'	110.8 (15)	N4—C26—C27	116.2 (5)
C34'—N5—C35'	124.7 (16)	N4—C26—H26A	108.2
C33—N5—C35	111.0 (15)	C27—C26—H26A	108.2
C33'—N5—C35	167.6 (15)	N4—C26—H26B	108.2
C34'—N5—C35	47.9 (11)	C27—C26—H26B	108.2
C34—N5—C35	118.2 (14)	H26A—C26—H26B	107.4
C35'—N5—C35	80.2 (14)	C32—C27—C28	118.7 (7)
Ni1—O1—H1C	110.9	C32—C27—C26	120.4 (8)
Ni1—O1—H1D	111.1	C28—C27—C26	121.0 (7)
H1C—O1—H1D	109.1	C27—C28—C29	119.0 (9)
Ni2—O2—H2C	107.3	C27—C28—H28	120.5
Ni2—O2—H2D	109.3	C29—C28—H28	120.5
H2C—O2—H2D	107.9	C30—C29—C28	118.6 (10)
C33'—O3—C33	62.6 (13)	C30—C29—H29	120.7
N1—C1—C2	109.0 (5)	C28—C29—H29	120.7
N1—C1—H1A	109.9	C31—C30—C29	123.5 (8)
C2—C1—H1A	109.9	C31—C30—H30	118.2
N1—C1—H1B	109.9	C29—C30—H30	118.2
C2—C1—H1B	109.9	C30—C31—C32	116.1 (9)
H1A—C1—H1B	108.3	C30—C31—H31	121.9
N2—C2—C1	110.0 (5)	C32—C31—H31	121.9
N2—C2—H2A	109.7	C31—C32—C27	123.9 (9)
C1—C2—H2A	109.7	C31—C32—H32	118.0
N2—C2—H2B	109.7	C27—C32—H32	118.0
C1—C2—H2B	109.7	O3—C33—N5	119.8 (17)
H2A—C2—H2B	108.2	O3—C33—H33	120.1
N1—C3—C4	115.1 (5)	N5—C33—H33	120.1
N1—C3—H3A	108.5	N5—C34—H34A	109.5
C4—C3—H3A	108.5	N5—C34—H34B	109.5
N1—C3—H3B	108.5	N5—C34—H34C	109.5
C4—C3—H3B	108.5	N5—C35—H35A	109.5
H3A—C3—H3B	107.5	N5—C35—H35B	109.5
C5—C4—C9	116.4 (6)	N5—C35—H35C	109.5
C5—C4—C3	121.3 (6)	O3—C33'—N5	119.7 (17)
C9—C4—C3	122.4 (5)	O3—C33'—H33'	120.2
C4—C5—C6	121.7 (7)	N5—C33'—H33'	120.2
C4—C5—H5	119.1	N5—C34'—H34D	109.5
C6—C5—H5	119.1	N5—C34'—H34E	109.5
C7—C6—C5	120.2 (7)	H34D—C34'—H34E	109.5
C7—C6—H6	119.9	N5—C34'—H34F	109.5
C5—C6—H6	119.9	H34D—C34'—H34F	109.5
C8—C7—C6	120.2 (7)	H34E—C34'—H34F	109.5
C8—C7—H7	119.9	N5—C35'—H35D	109.5
C6—C7—H7	119.9	N5—C35'—H35E	109.5
C7—C8—C9	118.7 (7)	H35D—C35'—H35E	109.5
C7—C8—H8	120.6	N5—C35'—H35F	109.5
C9—C8—H8	120.6	H35D—C35'—H35F	109.5
C4—C9—C8	122.8 (6)	H35E—C35'—H35F	109.5
C4—C9—H9	118.6		
			
O1—Ni1—N1—C3	−44.8 (4)	N2—C10—C11—C16	−69.1 (9)
O1^i^—Ni1—N1—C3	135.2 (4)	N2—C10—C11—C12	109.3 (7)
N2^i^—Ni1—N1—C3	40.2 (4)	C16—C11—C12—C13	−0.1 (12)
N2—Ni1—N1—C3	−139.8 (4)	C10—C11—C12—C13	−178.5 (7)
O1—Ni1—N1—C1	81.3 (3)	C11—C12—C13—C14	0.6 (13)
O1^i^—Ni1—N1—C1	−98.7 (3)	C12—C13—C14—C15	−1.8 (15)
N2^i^—Ni1—N1—C1	166.2 (3)	C13—C14—C15—C16	2.5 (16)
N2—Ni1—N1—C1	−13.8 (3)	C12—C11—C16—C15	0.7 (12)
O1—Ni1—N2—C2	−106.3 (3)	C10—C11—C16—C15	179.2 (7)
O1^i^—Ni1—N2—C2	73.7 (3)	C14—C15—C16—C11	−1.9 (14)
N1^i^—Ni1—N2—C2	164.2 (3)	C19—N3—C17—C18	95.7 (6)
N1—Ni1—N2—C2	−15.8 (3)	Ni2—N3—C17—C18	−34.8 (6)
O1—Ni1—N2—C10	20.8 (4)	C26—N4—C18—C17	−168.3 (5)
O1^i^—Ni1—N2—C10	−159.2 (4)	Ni2—N4—C18—C17	−41.5 (6)
N1^i^—Ni1—N2—C10	−68.6 (4)	N3—C17—C18—N4	53.6 (7)
N1—Ni1—N2—C10	111.4 (4)	C17—N3—C19—C20	66.8 (7)
O2^ii^—Ni2—N3—C19	149.0 (4)	Ni2—N3—C19—C20	−168.6 (4)
O2—Ni2—N3—C19	−31.0 (4)	N3—C19—C20—C25	−143.9 (7)
N4—Ni2—N3—C19	−119.5 (4)	N3—C19—C20—C21	41.4 (10)
N4^ii^—Ni2—N3—C19	60.5 (4)	C25—C20—C21—C22	1.4 (11)
O2^ii^—Ni2—N3—C17	−82.4 (4)	C19—C20—C21—C22	176.3 (7)
O2—Ni2—N3—C17	97.6 (4)	C20—C21—C22—C23	−0.2 (13)
N4—Ni2—N3—C17	9.1 (4)	C21—C22—C23—C24	−1.8 (13)
N4^ii^—Ni2—N3—C17	−170.9 (4)	C22—C23—C24—C25	2.5 (13)
O2^ii^—Ni2—N4—C18	107.4 (4)	C21—C20—C25—C24	−0.7 (11)
O2—Ni2—N4—C18	−72.6 (4)	C19—C20—C25—C24	−175.7 (7)
N3—Ni2—N4—C18	17.1 (4)	C23—C24—C25—C20	−1.2 (13)
N3^ii^—Ni2—N4—C18	−162.9 (4)	C18—N4—C26—C27	−63.0 (8)
O2^ii^—Ni2—N4—C26	−131.0 (4)	Ni2—N4—C26—C27	176.8 (5)
O2—Ni2—N4—C26	49.0 (4)	N4—C26—C27—C32	112.1 (9)
N3—Ni2—N4—C26	138.7 (4)	N4—C26—C27—C28	−68.1 (11)
N3^ii^—Ni2—N4—C26	−41.3 (4)	C32—C27—C28—C29	3.7 (14)
C3—N1—C1—C2	172.3 (5)	C26—C27—C28—C29	−176.1 (8)
Ni1—N1—C1—C2	41.3 (5)	C27—C28—C29—C30	−3.1 (15)
C10—N2—C2—C1	−85.9 (6)	C28—C29—C30—C31	0.7 (16)
Ni1—N2—C2—C1	43.6 (5)	C29—C30—C31—C32	1.1 (16)
N1—C1—C2—N2	−59.5 (6)	C30—C31—C32—C27	−0.5 (15)
C1—N1—C3—C4	55.2 (7)	C28—C27—C32—C31	−1.9 (15)
Ni1—N1—C3—C4	178.4 (4)	C26—C27—C32—C31	177.8 (8)
N1—C3—C4—C5	−133.1 (7)	C33'—O3—C33—N5	1.8 (17)
N1—C3—C4—C9	48.3 (9)	C33'—N5—C33—O3	−1.7 (16)
C9—C4—C5—C6	−0.4 (11)	C34'—N5—C33—O3	166 (2)
C3—C4—C5—C6	−179.1 (7)	C34—N5—C33—O3	0(3)
C4—C5—C6—C7	−1.3 (13)	C35'—N5—C33—O3	−3(10)
C5—C6—C7—C8	1.7 (13)	C35—N5—C33—O3	−175.5 (17)
C6—C7—C8—C9	−0.3 (12)	C33—O3—C33'—N5	−1.8 (17)
C5—C4—C9—C8	1.9 (10)	C33—N5—C33'—O3	1.7 (17)
C3—C4—C9—C8	−179.4 (6)	C34'—N5—C33'—O3	−11 (3)
C7—C8—C9—C4	−1.5 (11)	C34—N5—C33'—O3	−177 (2)
C2—N2—C10—C11	−53.8 (7)	C35'—N5—C33'—O3	−179 (2)
Ni1—N2—C10—C11	−176.8 (5)	C35—N5—C33'—O3	30 (8)

Symmetry codes: (i) −*x*+1, −*y*+1, −*z*+2; (ii) −*x*+1, −*y*+1, −*z*+1.

### Hydrogen-bond geometry (Å, °)

**Table:**

*D*—H···*A*	*D*—H	H···*A*	*D*···*A*	*D*—H···*A*
O1—H1C···Cl2	0.85	2.26	3.055 (4)	155
O1—H1D···Cl1^ii^	0.85	2.24	3.078 (4)	169
O2—H2C···Cl1^ii^	0.85	2.24	3.071 (4)	168
O2—H2D···Cl2	0.85	2.19	3.041 (4)	174
N1—H1···Cl1^iii^	0.91	2.54	3.444 (4)	171
N2—H2···O3^i^	0.91	2.20	3.102 (8)	169
N3—H3···Cl1	0.91	2.55	3.400 (4)	155
N4—H4···Cl2^ii^	0.91	2.54	3.428 (5)	167

Symmetry codes: (ii) −*x*+1, −*y*+1, −*z*+1; (iii) *x*, *y*, *z*+1; (i) −*x*+1, −*y*+1, −*z*+2.
